# Frequency of influenza H3N2 intra-subtype reassortment: attributes and implications of reassortant spread

**DOI:** 10.1186/s12915-016-0337-3

**Published:** 2016-12-29

**Authors:** Irina Maljkovic Berry, Melanie C. Melendrez, Tao Li, Anthony W. Hawksworth, Gary T. Brice, Patrick J. Blair, Eric S. Halsey, Maya Williams, Stefan Fernandez, In-Kyu Yoon, Leslie D. Edwards, Robert Kuschner, Xiaoxu Lin, Stephen J. Thomas, Richard G. Jarman

**Affiliations:** 1 Walter Reed Army Institute of Research, Silver Spring, MD USA; 2 Operational Infectious Diseases Directorate, Naval Health Research Center, San Diego, CA USA; 3 US Naval Medical Research Unit −6, Lima, Peru; 4 Armed Forces Research Institute of Medical Sciences, Bangkok, Thailand; 5 Office of Medical Services, US Department of State, Washington, DC USA; 6 Present Address: International Vaccine Institute, Seoul, Republic of Korea

**Keywords:** Influenza, Reassortment, H3N2, Spread

## Abstract

**Background:**

Increasing evidence suggests that influenza reassortment not only contributes to the emergence of new human pandemics but also plays an important role in seasonal influenza epidemics, disease severity, evolution, and vaccine efficacy. We studied this process within 2091 H3N2 full genomes utilizing a combination of the latest reassortment detection tools and more conventional phylogenetic analyses.

**Results:**

We found that the amount of H3N2 intra-subtype reassortment depended on the number of sampled genomes, occurred with a steady frequency of 3.35%, and was not affected by the geographical origins, evolutionary patterns, or previous reassortment history of the virus. We identified both single reassortant genomes and reassortant clades, each clade representing one reassortment event followed by successful spread of the reassorted variant in the human population. It was this spread that was mainly responsible for the observed high presence of H3N2 intra-subtype reassortant genomes. The successfully spread variants were generally sampled within one year of their formation, highlighting the risk of their rapid spread but also presenting an opportunity for their rapid detection. Simultaneous spread of several different reassortant lineages was observed, and despite their limited average lifetime, second and third generation reassortment was detected, as well as reassortment between viruses belonging to different vaccine-associated clades, likely displaying differing antigenic properties. Some of the spreading reassortants remained confined to certain geographical regions, while others, sharing common properties in amino acid positions of the HA, NA, and PB2 segments, were found throughout the world.

**Conclusions:**

Detailed surveillance of seasonal influenza reassortment patterns and variant properties may provide unique information needed for prediction of spread and construction of future influenza vaccines.

**Electronic supplementary material:**

The online version of this article (doi:10.1186/s12915-016-0337-3) contains supplementary material, which is available to authorized users.

## Background

Influenza is one of the most important human respiratory infections, and its seasonal recurrence is a major contributor to human morbidity and mortality. Seasonal influenza has an estimated annual attack rate of 10–20%, leading to 3–5 million cases of severe illness and 250,000 to 500,000 deaths each year [1]. Throughout history, influenza A has been the greatest contributor to human pandemics. During the last century, four influenza A pandemics (1918, 1957, 1968, and 2009) caused more than 50 million deaths globally, created significant social and economic impact on the human society, and shaped future research and public health policies.

The emergence and evolution of pandemic influenza has been greatly shaped by the process of segment reassortment occurring between different influenza subtypes, a process that enables swift changes of viral antigenic properties and may generate variants to which there is little to no immunity in the general population [2–4]. In addition, the mechanism of reassortment significantly contributes to the overall genetic and antigenic variability of the virus, substantially increasing its ability to quickly adapt to changing environments [5–7]. However, the process of inter-subtype reassortment may be restricted by substantial segment incompatibility, resulting in lower viral fitness and in limited success of reassortant spread in the human population [8, 9]. Indeed, despite high estimates of influenza reassortment capability, relatively few inter-subtype reassortants have actually established sustained human infections [7, 10–13].

Unlike inter-subtype reassortment, reassortment between different lineages of the same influenza subtype (intra-subtype) may not be as restricted due to higher genetic relatedness and functional compatibility of their segments [14, 15]. Like inter-subtype reassortment, however, intra-subtype reassortment plays a crucial role in increasing viral genetic diversity and adaptation plasticity [16–20]. The importance and the impact of influenza intra-subtype reassortment extend to all aspects of the influenza virus, from its evolution to its spread, disease severity, and vaccine efficacy. For instance, reassortment events within the influenza H1N1 subtype triggered the emergence of unusually severe seasonal epidemics of 1947 and 1951, and reassortment within the seasonal H3N2 influenza led to the dramatic rise and worldwide spread of resistance to adamantine drugs [21, 22]. In 2003, reassortment between two antigenically distinct lineages of H3N2 caused a major change in viral antigenic properties, leading to poor vaccine match and reduced vaccine effectiveness [23, 24]. Intra-subtype reassortment of H3N2 has also been shown to temporarily increase the rate of adaptive amino acid replacements within the reassorted variants and to be the major determinant of this virus’s evolution, superseding the contribution of adaptive processes caused by drift [17, 18, 20, 23, 25, 26].

As the role of influenza virus intra-subtype reassortment becomes increasingly apparent, identification and surveillance of its occurrence become ever more important. In general, detection of influenza virus reassortment requires screening of segment phylogenetic trees, where tree incongruences point to differences in segment evolutionary history. Until recently, intra-subtype reassortment has been studied by manual comparisons of segment phylogenies, a highly time-consuming task imposing restrictions on the size of the data and the depth and detail of the analyses. In addition, higher relatedness of sequences from the same subtype leads to a less pronounced phylogenetic signal, making detection of recent and subtle reassortments particularly challenging [21, 23, 27, 28]. The latest developments in automated tools for detection of influenza reassortment have allowed for bigger and more thorough analyses and, by accounting for uncertainties in inferred phylogenies, have also made the identification of both inter- and intra-subtype reassortment a more robust process [10, 18, 20, 25, 28–30]. This has contributed to our understanding of the occurrence and the importance of influenza intra-subtype reassortment; however, a clear picture of influenza intra-subtype reassortment and its influence throughout the world is still missing. In this study, we utilize the latest advanced approaches in combination with more conventional phylogenetic analyses to determine the frequency of H3N2 intra-subtype reassortment in the human population, as well as to investigate its implications on virus evolution, transmissibility, spread, and vaccine efficacy and prediction.

## Methods

### Sample collection, sequencing, and genome construction

Following combined surveillance efforts by the Department of State (DoS), Armed Forces Research Institute of Medical Sciences (AFRIMS), Naval Health Research Center (NHRC), Naval Medical Research Unit 6 (NAMRU-6), United States Air Force School of Aerospace Medicine (USAFSAM), and Walter Reed Army Institute of Research (WRAIR), 151 H3N2 influenza nasal or throat swabs were collected following Influenza-Like Illness (ILI) surveillance protocols. The samples were collected from different locations of the world between years 2009 and 2014. Primary isolates were made in Madin-Darby canine kidney (MDCK) cells either at WRAIR or in a host country laboratory as part of standard diagnostic procedures. All the samples and isolates were sent to WRAIR where viral RNA was extracted using QIAamp Viral RNA Mini Kit (Qiagen, Cat. number 52906).

For samples acquired before 2012, whole genome complementary DNA (cDNA) was generated using RT-PCR, and DNA was amplified by nested PCR using 48 pairs of fusion primers (H3N2-specific) with 454 adaptors and barcodes attached to the 5’ ends (Additional file 1). DNA was purified using the QIAquick PCR Purification Kit (Qiagen, Cat. number 28106) and quantified using an Agilent DNA1000 chip. An equal amount, 500 ng in total, of DNA of each sample (up to 96 samples) was pooled together for downstream rapid library preparation, emulsion PCR amplification (Lib-L, Large volume kit), and titanium sequencing (Roche 454 FLX + system), following manufacturers’ protocols.

For samples acquired between 2012 and May 2014, whole genome viral RNA was translated into first strand cDNA using SuperScript III reverse transcriptase (Invitrogen, Cat. number 18080044), and the DNA amplification was performed using the Platinum Taq DNA Polymerase High Fidelity System (Invitrogen, Cat. number 11304–102) according to manufacturers’ protocols. A single pair of universal primers and eight pairs of segment-specific primers were used for the two-step RT-PCR (Additional file 1). PCR products were loaded onto 1.0% agarose gel and electrophoresis was performed to check DNA quality. Segments for the same samples were pooled together, and the DNA was purified using the QIAquick PCR Purification Kit (Qiagen, Cat. number 28106). 500 ng of each sample was sonicated, and the 454 adaptor and barcode were ligated before proceeding to rapid library preparation, emulsion PCR amplification, and titanium sequencing (Roche 454 FLX + system). Gaps and low sequence coverage were filled by Sanger sequencing using the BigDye Terminator v3.1 Cycle Sequencing Kit (ABI, 4337456) on the ABI 3130XL Genetic Analyzer. For 9 samples collected between 2012 and 2014, the purified DNA was quantified with a NanoDrop 1000 Spectrophotometer, and 1 μg of each sample was fragmented using a Covaris S2 sonicator. Libraries with unique barcodes were made with the Ion Plus Fragment Library Kit (ABI, Cat. number 4471252) following manufacturer protocols. Libraries were quantified and pooled together for emulsion PCR using the Ion One Touch 200 Template Kit (ABI, Cat. number 4478316). Enriched template-positive libraries were sequenced using the Ion PGM 200 Sequencing Kit (ABI, Cat. number 4474004) and an Ion 316 Chip.

For samples acquired after May 2014, two-step RT-PCR was performed as described above using the same primers (Additional file 1). PCR products were loaded onto 1.0% agarose gel and electrophoresis was performed to check DNA quality. Segments for the same samples were pooled together, and the DNA was purified using the QIAquick PCR Purification Kit (Qiagen, Cat. number 28106). Purified DNA was quantified using the Quant-iT PicoGreen dsDNA Assay Kit (Invitrogen, Cat. number P11496). The DNA in each sample was diluted to 0.2 ng/μl prior to library preparation based on its PicoGreen reading. Following the manufacturer’s protocol, 5 μl of DNA (1 ng per sample) was used as start material for library preparation (Nextera XT DNA Sample Preparation Kit, Cat. number FC-131-1096). Libraries were normalized, pooled together, and loaded onto the MiSeq sequencing reaction cartridge (MiSeq Reagent kit v2, 500 cycles, Cat. number MS-102-2003) for sequencing. Genomes were constructed from raw fastq and sff files generated by the sequencing instruments using the ngs_mapper v1.2, an in-house developed reference mapping pipeline [31]. All genomes were submitted to GenBank under accession numbers [GenBank:KT888068]–[Genbank:KT889275].

### Sequence dataset compilation

In April 2014, all available, complete genome sequences of influenza H3N2, sampled between 2008 and 2013, were downloaded from the Influenza Virus Resource (IVR), the Influenza Research Database, and the Global Initiative on Sharing All Influenza Data (GISAID) EpiFlu^TM^ Database [32–34]. This also included 18 full genome vaccine and vaccine candidate sequences sampled between 2005 and 2013, 11 full genome sequences belonging to the CA04 and PE09 antigenic clusters [20], and 5 full genome sequences sampled between 1977 and 1987 (to be used as an outgroup). No full genome sequences were available at that time point for year 2014 in any of the databases. The quality of the obtained sequences was evaluated using several steps, including the IVR annotation tool, alignment, and phylogenetic inference; sequences with unexpected stop codons, consecutive Ns, unusual frame shifts and indels, and those with very long branches in trees were removed from the dataset. This high-quality dataset comprised 2154 database-derived full genomes with known sampling dates and locations. From these, the H3N2v sequences [35, 36] were removed, resulting in a dataset of 1940 full genomes. All WRAIR and downloaded sequences were pooled into one single dataset, comprising a total of 2091 H3N2 full genomes (accession numbers for reference genomes are listed in Additional file 2).

### Phylogenetic inference

Separate sequence alignments were made for all 8 influenza segments, containing all 2091 sequences each, using MAFFT v7 and manually examined in MEGA v6 [37, 38]. In addition, a full genome alignment was compiled by concatenation of all 8 segments. The best-fit model of evolution for each of the segments was determined using jModelTest v2.1.7 based on the Akaike information criterion (AIC) [39]. Maximum likelihood (ML) phylogenetic trees for each of the segments, and for the full genome, were inferred using PhyML v4.9.1 [40, 41]. The model of evolution used for the full genome tree inference was GTR+I+Γ (general time reversible with empirically estimated proportion of invariant sites and gamma distribution of among-site variation, 4 categories) [42–44], as it was the most complex model found for some of the segments. The tree space was searched heuristically using the best of NNI (nearest neighbor interchanges) and SPR (subtree pruning and regrafting). Node confidence values were determined by aLRT (approximate likelihood-ratio test) using the non-parametric Shimodaira-Hasegawa approach [45]. All trees were rooted by the 5 outgroup genomes (sampled 1977–1987). In addition, all vaccine genomes, genomes belonging to a known antigenic cluster (CA04 and PE09) and genomes sampled between 1977 and 1987 were removed from the full dataset (*N* = 34). This reduced dataset (*N* = 2057) was randomly subsampled 20 times creating 20 smaller data subsets, containing between 251 and 261 randomly sampled genomes. To each of these, all the 34 removed genomes were added back, to serve as a consistent reference in downstream analyses. The 20 subsets comprised 8 segment alignments each. The Metropolis-coupled Markov chain Monte Carlo (MCMC) algorithm with 2 runs and 4 chains, as implemented in MrBayes v3.2 [46], was used to sample trees for all 160 alignments. The chains were run for 50 million generations, with a burn-in of 25% and a sampling frequency of 25,000, utilizing evolutionary models previously estimated by jModelTest.

### Reassortment detection

For every one of the 20 data subsets used in MrBayes, all segment tree files, with 2000 sampled trees each, were compared and searched for phylogenetic incongruences and reassortment events using a biclique enumeration algorithm with phylogenetic discordance test, as implemented in the Graph-incompatibility-based Reassortment Finder (GiRaF) v1.02 [28]. The burn-in was set to 25%, and resulting reassortment events with confidence levels >0.9 were considered accurate. The 34 shared reference genomes recurring in each of the data subsets, together with some repeatedly randomly sampled sequences, also aided in confirmation of reassortant groups/events. However, we were aware that some of the genomes from the big dataset (*N* = 2057) might not have been sampled by chance, and their possible involvement in reassortment would remain unresolved. Thus, all reassortants were mapped onto the maximum likelihood (ML) segment and full genome phylogenetic trees (inferred by using all 2091 genomes) and visualized using FigTree [47]. Manual investigation of the genome movement across the segment ML trees allowed for confirmation or rejection of complex and unresolved reassortment patterns found by GiRaF. Investigation of the full genome ML tree resulted in a more confident assignment of genomes not randomly sampled in any of our 20 data subsets and genomes not properly assigned because of low phylogenetic signal, as well as final identification of reassortant clades representing viruses generated from the same reassortment event (designated as lineages *I-XVI*). For instance, as all reassortant genomes belonging to a group found by MrBayes/GiRaF analyses clustered together in a highly supported clade in the full genome tree, any unassigned genomes also found within this clade could correctly be categorized as belonging to the same reassortment event. Thus, a reassortant clade/lineage was defined as the smallest clade within which all identified reassortants were found. In addition, manual investigation of the full dataset segment ML trees (Additional files 3, and 4) allowed for identification of false positive reassortant clades. Because GiRaF determines reassortment events by detecting incongruences between the segment trees, reassortant groups/clades will be detected if they occupy different positions in different segment trees. However, movement of one clade can result in displacement of another clade in a tree, without the latter one being a true reassortant. Careful examination of the segment phylogenetic trees revealed this to be the case, and the false positive reassortants detected by GiRaF were removed from the results. In the end, only reassortants confirmed by both MrBayes/GiRaF and ML tree inspections were accepted.

### RDP analyses

Recombination Detection Program version 4 (RDP4) [48] was used to confirm reassortment events detected using MrBayes/GiRaF and ML. Given that we were specifically investigating full segment reassortment events rather than intra-segment recombination, the concatenated full genomes were constructed and the recombination events were required to be significantly supported (*p* value <0.05) by at least 3 of the 8 methods employed within the RDP4 package. To minimize computational burden and allow for timely recombination analyses, all sequences belonging to the GiRaF-detected reassortant clades/lineages were removed from the alignment, and their ancestral sequences, inferred by the joint ancestral sequence reconstruction (ASR) in HyPhy [49] (reassortants of reassortants not included), were added instead.

### Reassortment frequency

The frequency of H3N2 intra-subtype reassortment for 2008–2014 was calculated for both the 20 separate data subsets generated by the MrBayes/GiRaF analyses and for the dataset as a whole generated by a combination of MrBayes/GiRaF and ML analyses. The frequency of reassortment events was estimated as the total number of reassortant groups/events and unique reassortants, divided by the number of all genomes. The fraction of reassortant genomes was estimated as the total number of reassortant genomes divided by the number of all genomes. The 34 recurring genomes were not included in these calculations.

Evaluation of continuous reassortment was performed on 15 of our identified reassortant clades/lineages, excluding lineage *II* because of its association with regional and temporal sampling limitations. Instead, the large Texas/50/2012 vaccine-associated clade/lineage was included, as it contained the most recently sampled sequences and was of non-reassortant origin. Frequency of continued reassortment was calculated as the number of reassortment events within a lineage divided by the total number of genomes within that lineage/clade. The number of reassortment events was weighted to correctly estimate the contribution of inter-lineage reassortant genomes. All reassortant genomes with all of the segments originating from within a lineage contributed with a score of 1 for that lineage. Inter-lineage reassortants contributed with a score of 1 divided by the number of lineages from which their segments were derived. Thus, if a lineage had 3 within-lineage reassortants, and an additional 2 reassortants with some segments originating from that lineage and the rest from another one, the total reassortment score for the lineage in question would be 3 + 1/2 + 1/2 = 4.

For comparison of reassortment frequencies between different reassortant and/or non-reassortant clades, we used z-test. However, z-test could not be used on clades that consisted of very few genomes and where this frequency was 0. Thus, we performed a chi-squared test comparing the number of observed reassortment events to the number of expected reassortment events given the previously estimated reassortment frequency of 3.35%.

Adjustment for sampling bias towards North America, where approximately half of the genomes originated, was made by randomly subsampling the North American dataset 10 times such that each of the 10 data subsets contained 300 genomes each from this region. The full dataset (*N* = 2091) was stripped from all North American genomes, and this reduced set was added to the 10 North American random subsets. The frequency of reassortment events and the fraction of the reassortant genomes were calculated for each of the subsets as described above.

### Timing of reassortment events

Bayesian Evolutionary Analysis Sampling Trees (BEAST v1.8) [50] was used to estimate the time of formation of the 16 reassortant lineages/clades. Because of the size of the full dataset, which was too large for timely analyses with BEAST, three smaller datasets were constructed that included all reassortant genomes belonging to the groups/clades of interest and 200 randomly sampled sequences from the rest of the data. The 5 genomes sampled between 1977 and 1987 were also included. The first dataset (BEASTA) consisted of 359 sequences from the reassortant clade *X* and 200 randomly sampled sequences (*N* = 559), the second dataset (BEASTE) included 183 sequences from the reassortant clades *II, III, XI*, and *XII* and 200 randomly sampled sequences (*N* = 388), and the third dataset (BEASTF) had 216 sequences from the reassortant clades *I, IV, V, VI, VII, VIII, IX, XIII, XIV, XV*, and *XVI* and 200 randomly sampled sequences (*N* = 421). All 8 segments from the three smaller datasets were analyzed. The Markov Chain Monte Carlo (MCMC) chains were run for 500 million generations, sampling every 50,000, with a strict clock and Bayesian skyride with Gaussian Markov Random Field (GMRF) smoothing prior. Models of nucleotide evolution were used as estimated by jModelTest v2.1.7. In cases where the resulting evolutionarily stable strategy (ESS) values were low (<200), we preformed three separate MCMC runs and combined the results. In three instances, the strict clock had to be replaced by an exponential or lognormal one to achieve convergence. All the XML files (without sequence data due to GISAID restriction of data sharing) and a table of seeds for each run are available in Additional file 5. The time of creation of a certain reassortant was determined by the time of the most recent common ancestor (TMRCA) of its youngest segment, i.e., the segment with the most recent genetic sweep [18]. The segment TMRCA differences of a lineage were also used to confirm the reassortant nature of that lineage. In addition, the three BEAST datasets described above were used for (1) confirmation and reassortment pattern determination of the reassortant lineages by assigning lineage-specific discrete traits to the genomes comprising these lineages [10] and (2) phylogeographic analyses where discrete traits were represented by the region of sampling of a genome. However, these datasets proved too big for confident and timely discrete trait analyses and provided only fragmented information.

### Amino acid and entropy analyses in global and local lineages

In order to investigate differences between the 16 different reassortant clades/lineages, we divided them into different groups based on the following criteria. (1) Globally spread lineages (Global) were those where genomes were sampled from at least four different regions of the world and where one region contributed with less than 80% of the total number of sampled reassortant genomes. (2) Locally spread lineages were those where 80% or more of the reassortant genomes were sampled from the same region. Locally spread lineages were further divided into high (High Local) or low (Low Local), where High Local variants were the ones represented by >10% of *all* samples from that region. Using these criteria, the reassortant lineage/clade *II* was assigned High Local; however, it was excluded from the final comparison analyses as it contained the only sequences (locally sampled) available for year 2014. Because of uneven sampling of the downloaded influenza genomes, we assigned our geographical distribution with low resolution, and the regions were defined on continent geographic scale as North (N) America, South-Central (SC) America, Europe, Asia, Australia and Oceania, and Middle (M) East. Raw amino acid differences were compared for all viral proteins (HA, M1 and M2, NA, NP, NS1 and NS2, PA, PB1 and PB1_F2, and PB2) between Global and High Local reassortants, High Local and Low Local reassortants, and in between High Local reassortants from different regions of the world. Shannon entropy differences [51] were assessed for amino acid positions of all viral proteins between Global and High Local reassortants (pairwise comparisons) and in between High Local reassortants from different regions of the world. Low Locals were excluded because of low numbers resulting in low statistical power. Furthermore, to circumvent false negative results due to a low number of genomes in some of the tested reassortants, all Global reassortants were together compared to all High Local ones (grouped comparisons), and the positions found significant in both grouped and pairwise tests were examined for their amino acid compositions. Clade/lineage *II* was removed from the latter analyses.

### Selection analyses

In order to detect differences in positive selection between Global and High Local lineages, we investigated presence of positive selection in HA, NA, and PB2 coding regions of Global and High Local alignments separately, using several different approaches available in HyPhy: SLAC (Single Likelihood Ancestor Counting), FEL (Fixed Effects Likelihood), and FUBAR (Fast Unconstrained Bayesian Approximation) [49]. For SLAC and FEL, a *p* value <0.05 was used as significance cut-off, and for FUBAR a posterior probability >95 was used. Because certain nucleotide positions in some of our genomes consisted of International Union of Pure and Applied Chemistry (IUPAC) symbols for ambiguous nucleotides, and because HyPhy handles such cases as partially missing data, there was a risk of missing potentially important variation by not including the information found in the ambiguous positions. Thus, sequences with ambiguous nucleotide positions were duplicated and the positions in question changed to the existing variants as indicated by the IUPAC code, such that all existing original sequence variation was represented in the alignment. Outlier sequences (three from HA alignment and one from NA) with more than three ambiguous positions in each of the genes were not included in these analyses.

### Passaging analyses

In order to investigate whether passaging of isolates in MDCK affected our results for position NA 151, we collected all available passaging information from GenBank or GISAID for the reassortant isolates in this study. In 8 out of 13 local lineages the majority of isolates had available passaging history (Additional file 6). None of the global lineages contained enough viruses with available passaging information. However, one lineage that was predicted to be Global by our study, lineage *II*, did have enough passaging information and was thus used for statistical analyses against the Local lineages. Samples in each lineage were divided into two categories: not passaged + passaged in eggs (since egg passaging does not affect the composition of NA position 151) and passaged (including MDCK1, MDCK2, MDCK3, C1, C2, C3, RhMK1).

## Results

### Reassortment identification reveals the extent of its complexity

**Fig. 1:**
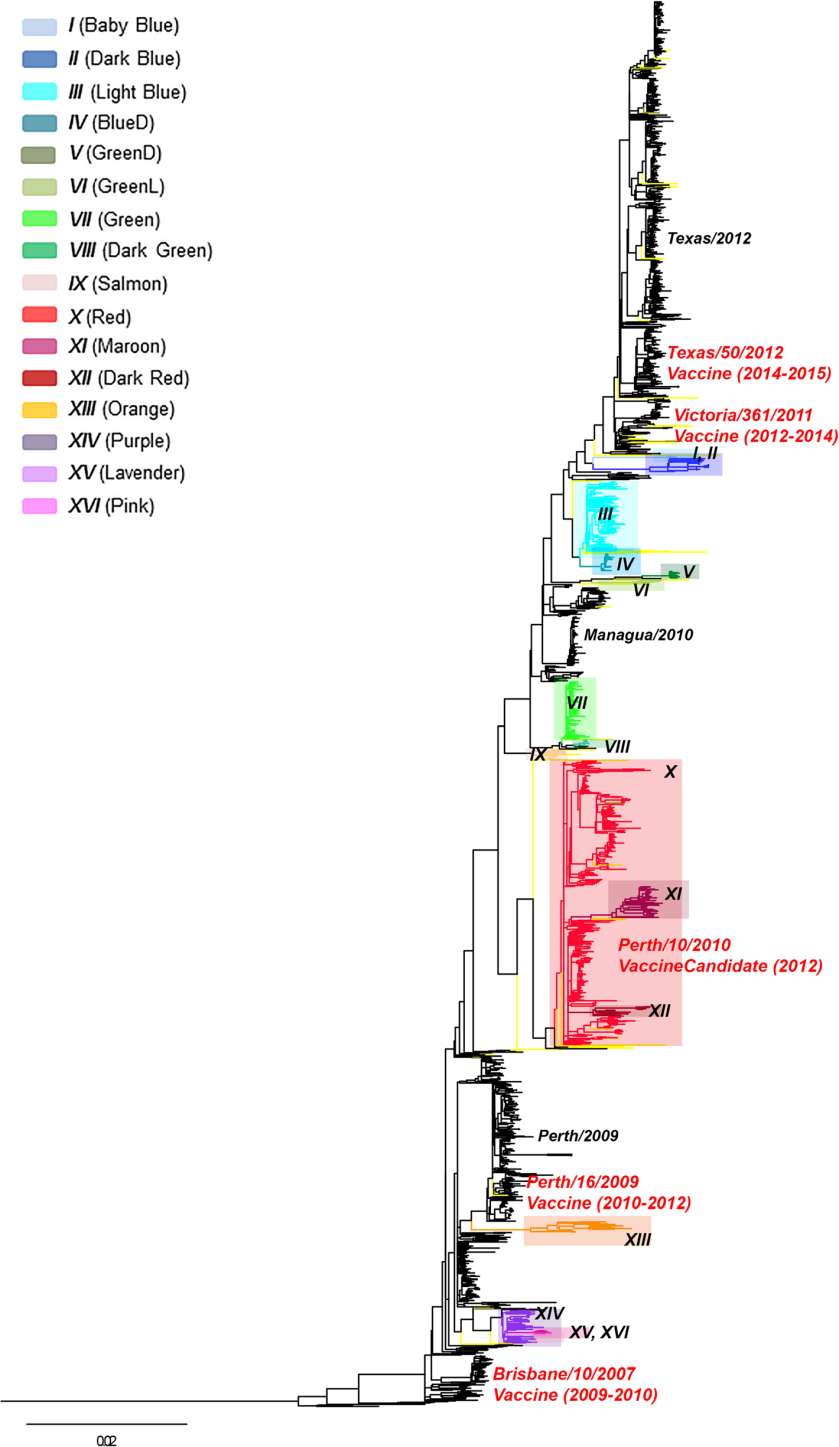
Reassortant lineages and variants found among H3N2 genomes sampled between 2008 and 2014. The full genome maximum likelihood phylogenetic tree shows 16 reassortant clades/lineages highlighted in Baby Blue (*I*), Dark Blue (*II*), Light Blue (*III*), BlueD (*IV)*, GreenD (*V*), GreenL (*VI*), Green (*VII*), Dark Green (*VIII*), Salmon (*IX*), Red (*X*), Maroon (*XI*), Dark Red (*XII*), Orange (*XIII*), Purple (*XIV*), Lavender (*XV*), and Pink (*XVI*) (in the order of appearance in the tree) and single reassortants marked in *yellow*. Vaccines and important vaccine candidate names are positioned next to their approximate position in the tree. Years of vaccine use are in parentheses next to the vaccine names. The three most distinct non-reassortant clades are named in *black* text. All reassortant and vaccine clades had node confidence values >0.7 (aLRT)

The dataset used in this study comprises 2091 H3N2 full genomes sampled from 56 different countries between 2008 and 2014. Identification of reassortment was performed by combined MrBayes/GiRaF and ML analyses of segment tree incongruence, where reassortant genomes found in MrBayes/GiRaF analyses were mapped onto the ML segment trees, and the results were used for confirmation of reassortment. Following this, the found reassortants were mapped onto a full genome ML tree, which was used for determination of reassortant lineages with reliance/confidence, and for further refined detection of genomes within these lineages (Fig. 1). There were a total of 16 reassortant clades/lineages found (*I* to *XVI*, Fig. 1, Table 1). Each of these lineages represented a single reassortment event followed by subsequent spread and sampling of the reassortant virus in the human population. Each of the reassortant lineages in the full genome ML tree (Fig. 1) was supported by high node confidence values (approximate likelihood-ratio test, aLRT >0.7). In several instances, one reassortant clade/lineage was found within another, indicating the presence of second-generation reassortment (lineages *XI, XII, XV*, and *XVI*). As noted previously, monophyly of reassortant clades was not always observed in the separate segment trees [25, 28].

**Table 1:** Reassortant geographical and temporal distributions and time of the most recent common ancestor (TMRCA)

Reassortant lineage	Genomes (*N*)^a^	Sampling year	TMRCA (95% HPD^b^)	Lineage persistence^c^ (CI)	Existence since TMRCA^d^(CI)	Sampling lag^g^	Global	High Local	Low Local
*I* (Baby Blue)	4	2012	2011.8 (2011.5-2011.9)	0.5 (0-1)*	0.7 (0.1-1.5)	0.2			N America
*II* (Dark Blue)^e^	20	2013-2014	2012.5 (2012.2-2012.8)	0.7 (0.53-0.87)	1.7 (1.23-2.17)	1		M East^e^	
*III* (Light Blue)	103	2010-2013	2009.7 (2009.6-2009.8)	3 (2.64-3.36)	3.8 (3.34-4.26)	0.8	Yes		
*IV* (BlueD)	25	2011	2010.3	0.5 (0-1)*	1.2 (0.7-1.7)	0.7		Asia	
*V* (GreenD)	11	2012	2011.5 (2011.2-2011.7)	0.5 (0-1)*	1 (0.3-1.8)	0.5			N America
*VI* (GreenL)	5	2012	2011.3 (2011-2011.7)	0.47 (0.27-0.67)	1.2 (0.6-1.7)	0.7		Australia	
*VII* (Green)	85	2010-2011	2009.2 (2009-2009.3)	1 (0.5-1.5)	2.3 (1.7-3)	1.3		SC America	
*VIII* (Dark Green)	8	2010	2009.7 (2009.4-2009.8)	0.5 (0-1)*	0.8 (0.2-1.6)	0.3			SC America
*IX* (Salmon)	13	2010	2009.3 (2009-2009.5)	0.5 (0-1)*	1.2 (0.5-2)	0.7			SC America
*X* (Red)	351	2010-2012	2008.8 (2008.3-2009.1)	2 (1.54-2.46)	3.7 (2.94-4.66)	1.7	Yes		
*XI* (Maroon)	46	2012-2013	2011.1 (2010.9-2011.3)	1 (0.5-1.5)	2.4 (1.7-3.1)	1.4		N America	
*XII* (Dark Red)	14	2011-2013	2010.9 (2010.6-2011.4)	2 (1.57-2.43)	2.6 (1.67-3.33)	0.6	Yes		
*XIII* (Orange)	15	2010-2011	2009.1 (2009-2009.2)	1 (0.5-1.5)	2.4 (1.8-3)	1.4		Asia	
*XIV* (Purple)	40	2009	2007.8 (2007.4-2008.1)	0.5 (0-1)*	1.7 (0.9-2.6)	1.2		N America	
*XV* (Lavender)	5	2009	2008.6 (2008.3-2008.9)	0.5 (0-1)*	0.9 (0.1-1.7)	0.4			N America
*XVI* (Pink)	3	2009	2008.9 (2008.7-2009)	0.5 (0-1)*	0.6 (0-1.3)	0.1			N America
Yellow (single unique)^f^	53 + 4	2008-2013							
Total	805								

^a^The 7 recurring reassortant vaccine genomes (see Methods) were not counted

^b^ *HPD* highest posterior density intervals

^c^Refers to the period of time when the lineage was observed in the human population

^d^Refers to the total lifespan of the reassortant variant in question

^e^Although assigned High Local by the criteria, it cannot with certainty be regarded as such due to low regional and temporal sampling

^f^4 genomes were identical to 4 of the unique reassortants

^g^Sampling lag is defined in the “Between-lineage and vaccine clade reassortment despite limited reassortant lifespan” subsection of Results

*All genomes were sampled in the same year with no exact dates. Thus, the persistence was given a value of 0.5, given the sampling uncertainty of 6 months

In addition to 16 reassortant clades/lineages, 53 genomes were found with unique reassortment patterns, and 4 additional genomes were identical to 4 of these unique reassortants. These genomes represented reassortant viruses that did not establish a successful spread in the human population. Some of these unique reassortants had segments derived from within the second-generation reassortant lineages, demonstrating the occurrence and detection of third-generation reassortment. Ten of the genomes showed more complex reassortment patterns of segment exchange, including segments originating from at least three different parental lineages/clades. Most, but not all, of the reassortants were also confirmed using RDP (Table 2). RDP is in general used to find parental genomes of recombinant sequences, and lack of their presence might have resulted in reassortment underestimation by this tool [48]. RDP also found a large amount of reassortment where none existed (169 false positive genomes), as compared by manual examination of the trees and GiRaF results. This has been observed in RDP previously [28].

**Table 2:** Ancestral sequences of the reassortant clades that were found recombinant by RDP4

Recombinant sequence(s)	Method								
	RDP [68]	GENECONV [69]	Bootscan [70]	Maxchi [71]	Chimera [72]	SiSscan [73]	PhylPro [74]	LARD [75]	3Seq [76]
*I* (Baby Blue_Ancestral)	NS	1.16E-02	0.026402	3.68E-06	4.39E-02	3.29E-05	NS	NS	5.77E-09
*II* (Dark Blue_Ancestral)^a^	4.92E-02	NS	4.95E-02	NS	NS	NS	NS	NS	NS
*VI* (GreenL_Ancestral)^a^	NS	NS	NS	1.17E-03	NS	NS	NS	NS	2.40E-02
*IX* (Salmon_Ancestral)	NS	NS	NS	3.02E-04	1.72E-04	8.99E-06	NS	NS	2.09E-11
*X* (Red_Ancestral)	1.81E-02	NS	1.82E-02	NS	NS	1.35E-02	NS	NS	NS
*XI* (Maroon_Ancestral)	1.81E-02	NS	1.82E-02	NS	NS	1.35E-02	NS	NS	NS
*XII* (Dark Red_Ancestral)	1.81E-02	NS	1.82E-02	NS	NS	1.35E-02	NS	NS	NS
*XIV* (Purple_Ancestral)	NS	NS	NS	2.00E-02	NS	9.47E-12	NS	NS	5.74E-04
*XV* (Lavender_Ancestral)	NS	NS	NS	2.00E-02	NS	9.47E-12	NS	NS	5.74E-04
*XVI* (Pink_Ancestral)	NS	NS	NS	2.00E-02	NS	9.47E-12	NS	NS	5.74E-04

^a^Trend of recombination, only 2 out of 3 required methods showed recombination

NS - not significant

Thus, we identified a total of 805 reassortant genomes in our dataset. These were a result of 69 different reassortment events, defined by 16 reassortant clades/lineages and 53 unique reassortants. We found the presence of both continued reassortment in the already reassorted lineages and of reassortment variants with their segments derived from three or more different parents.

### H3N2 intra-subtype reassortment exhibits stable event frequency

By dividing the number of reassortment events by the total number of influenza genomes, we estimated the frequency of H3N2 intra-subtype reassortment (during years 2008–2014) for both the 20 smaller data subsets used in MrBayes/GiRaF (MCMC) analyses, and for the full dataset derived by combined MCMC and ML analyses. The average frequency of reassortment events in the smaller data subsets was comparable to the frequency of the full dataset, 3.45% and 3.35%, respectively (Table 3a). The fraction of all the reassortant genomes in the full dataset was 39.1%, and in the 20 smaller subsets it averaged at 41.3% (Table 3a). We also observed high variation of frequency values between the individual small data subsets. This was a result of both (1) false positive reassortment events in some subsets, which resulted in reassortment overestimation, and (2) lack of phylogenetic signal for confident resolution of reassortment patterns in other subsets, resulting in reassortment underestimation. These results highlight the limitations of the small datasets (285–295 genomes) as well as the importance of secondary analyses for tree incongruence interpretations.

**Table 3:** Frequency of reassortment

Dataset	Total genomes (*N*)^a^	Reassortant genomes (*N*)	Fraction of reassortants	Reassortment events (*N*)	Event frequency
a) Frequency calculated on the 20 MrBayes + GiRaF subsets and the full dataset					
Subset 1	257	61	0.237	9	0.035
Subset 2	254	83	0.327	9	0.035
Subset 3	259	64	0.247	12	0.046
Subset 4	261	159	0.609	9	0.034
Subset 5	257	174	0.677	10	0.039
Subset 6	256	143	0.559	10	0.039
Subset 7	259	165	0.637	6	0.023
Subset 8	252	72	0.286	8	0.032
Subset 9	261	84	0.322	14	0.054
Subset 10	258	75	0.291	9	0.035
Subset 11	257	175	0.681	9	0.035
Subset 12	259	163	0.629	8	0.031
Subset 13	258	83	0.322	7	0.027
Subset 14	255	61	0.239	10	0.039
Subset 15	256	68	0.266	11	0.043
Subset 16	251	80	0.319	7	0.028
Subset 17	256	79	0.309	9	0.035
Subset 18	251	35	0.139	7	0.028
Subset 19	254	153	0.602	8	0.031
Subset 20	258	143	0.554	5	0.019
Average	256.45	106	0.4126	8.85	0.0345
SD			0.1752		0.0077
Full set	2057	805	0.3913	69	0.0335
b) Frequency calculated on the 10 data subsets adjusted for unequal sampling					
Subset 1	1188	619	0.521	36	0.030
Subset 2	1188	626	0.527	37	0.031
Subset 3	1188	629	0.529	38	0.032
Subset 4	1188	632	0.532	39	0.033
Subset 5	1188	631	0.531	41	0.035
Subset 6	1188	639	0.538	42	0.035
Subset 7	1188	632	0.532	37	0.031
Subset 8	1188	628	0.529	37	0.031
Subset 9	1188	633	0.533	36	0.030
Subset 10	1188	635	0.535	38	0.032
Average	1188	630.4	0.5306	38.1	0.0321

^a^Excluding the 34 recurring genomes

To further confirm the estimates of the H3N2 intra-subtype reassortment frequency, and to adjust for sampling bias towards North America in the full dataset, we subsampled genomes from North America such that their numbers approximately matched the numbers of genomes sampled in Asia and South America. Ten of these equalized datasets were made (Table 3b). The average frequency of reassortment events for the equalized datasets was comparable to the frequency of the full dataset (3.21% and 3.35%, respectively) and not significantly different from the frequency found in the 20 smaller MrBayes datasets (*t* test, *p* > 0.05). However, the fraction of total number of reassortant genomes in the equalized datasets was estimated at an average of 53.1%, significantly higher than the fraction in the MrBayes datasets (*t* test, *p* < 0.01).

**Fig. 2:**
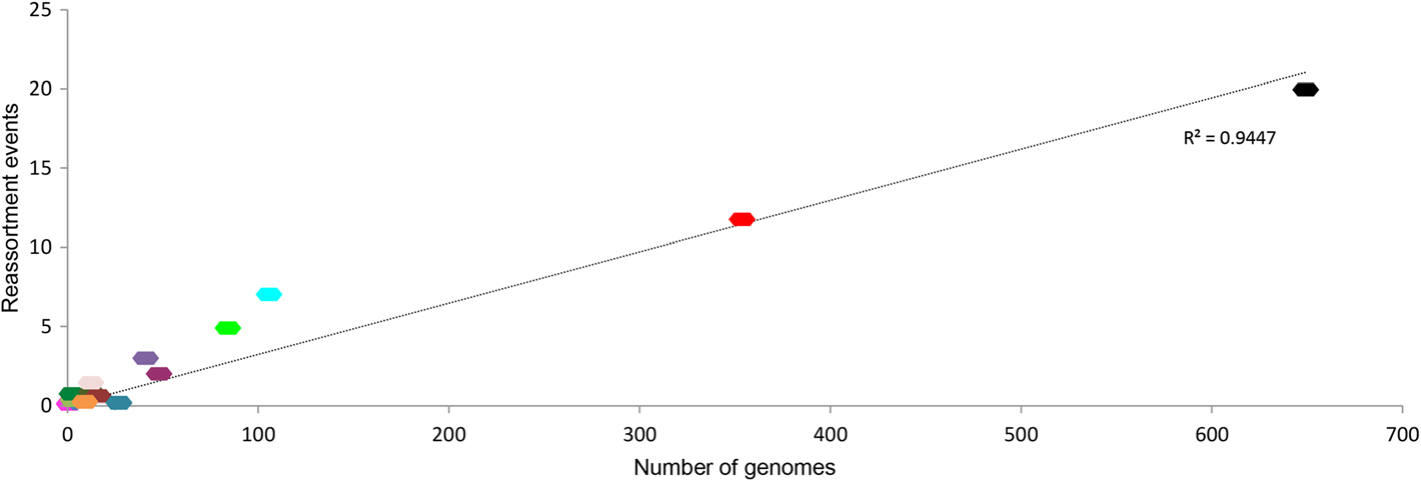
Reassortment frequency of the reassortant and non-reassortant lineages. The number of reassortment events in each of the lineages/clades as a function of the number of genomes belonging to that clade. Data points are highlighted in color of their reassortant lineage (see Fig. 1). The *top black* data point represents the Texas/50/2012 vaccine-associated non-reassortant clade. Inter-clade reassortment events were weighted

Since our data revealed the presence of second- and third-generation reassortment, we investigated whether viruses belonging to a certain reassortant clade/lineage continued reassorting more efficiently/often than viruses from other reassortant or non-reassortant clades. Analyses of 15 reassortant clades/lineages and 1 non-reassortant lineage revealed between 0 and 19.8 first-, second-, and third-generation reassortment events per lineage, with the number of events increasing with the lineage size (Spearman’s rank correlation, rho = 0.84, *p* < 0.01) (Fig. 2). The frequency of reassortment events did not differ significantly between the clades/lineages, regardless of lineage reassortment history, evolution, or geographical location, nor did it deviate significantly from the previously estimated frequency of 3.35% (z-test, *p* > 0.05; chi-squared test, *p* > 0.05).

Thus, the number of H3N2 intra-subtype reassortment events within a lineage depended on the number of genomes in that lineage and occurred with a steady frequency of 3.35%. It was not affected by the differences in lineage geographic backgrounds or their previous history of reassortment.

### Between-lineage and vaccine clade reassortment despite limited reassortant lifespan

Examination of sampling times of the genomes belonging to the 16 reassortant lineages revealed that all reassortant lineages did not exist simultaneously, but rather replaced each other over time. An average of 5.4 different reassortant lineages were found spreading during the same years, ranging from 3 in 2009 to 7 in 2010 and in 2012. The average lifespan of a reassortant lineage was 0.9 year, with some larger reassortant clades persisting 2–3 years (Table 1). Reassortant lineages spreading globally had a significantly higher persistence (2.33 years) than the locally spreading ones (0.63 year, *p* = 0.03, *t* test). In general, a reassortant was sampled in the year following the year of its formation, with an average of 0.81 year of lag time between reassortant formation and its sampling on the population level (sampling lag), and with no significant difference in lag time between globally and locally spreading lineages (*p* > 0.05, *t* test).

**Fig. 3:**
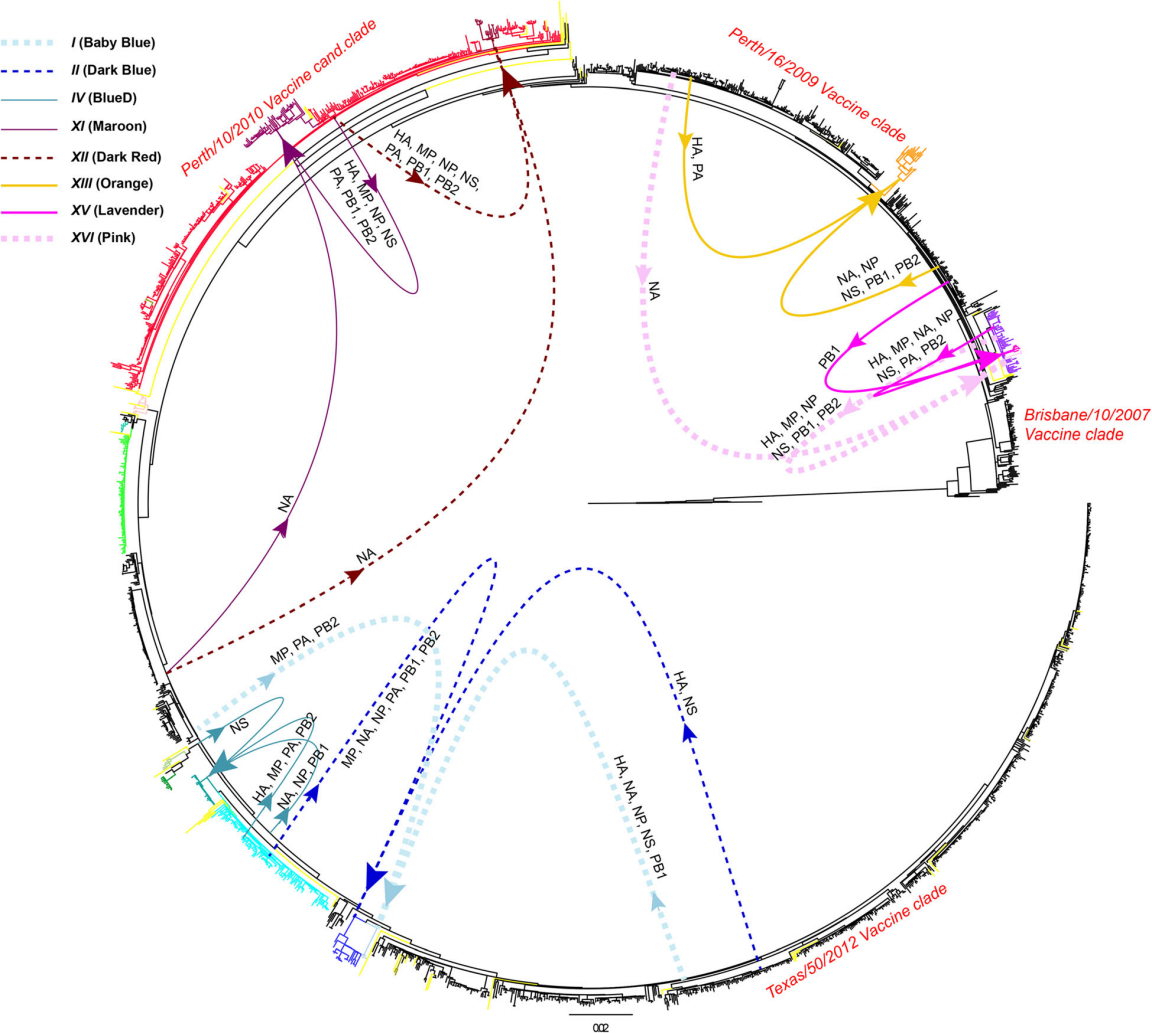
Reassortment origins and patterns of 8 reassortant lineages. Schematic figure of segment origins and patterns of intra-subtype reassortment of H3N2 reassortant lineages/clades. Each lineage represents one reassortment event. Approximate segment origins for 8 of the lineages are illustrated on a full genome maximum likelihood phylogenetic tree containing 2091 H3N2 genomes sampled between 2008 and 2014. Single unique reassortants are colored *yellow* in the tree. Origins and patterns were determined from ML and GiRaF analyses

**Fig. 4:**
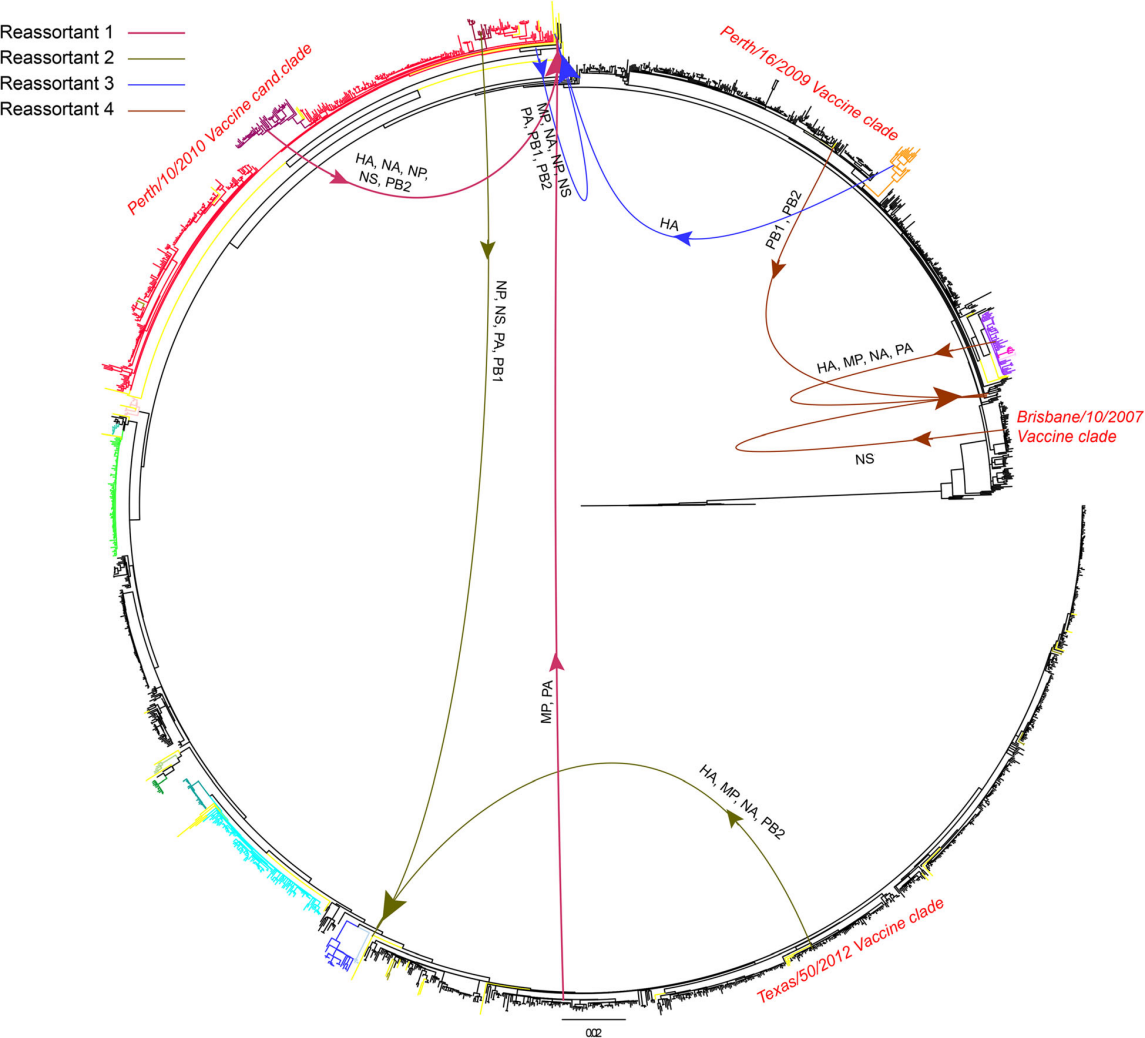
Reassortment origins and patterns of 4 genomes with segments from different vaccine-associated clades. Schematic figure of segment origins and patterns of intra-subtype reassortment of selected H3N2 reassortant genomes. Approximate segment origins are illustrated on a full genome maximum likelihood phylogenetic tree containing 2091 H3N2 genomes sampled between 2008 and 2014. Single unique reassortants are colored *yellow* in the tree. Origins and patterns were determined from ML and GiRaF analyses

The patterns of segment exchange differed vastly between the reassortant lineages. Origins of viral segments were generally easier to determine for between-lineage reassortants occurring later in time, and for those with segments originating from only two different parents. Although GiRaF performed well in identification of the patterns of segment exchange in these reassortants, we observed the occurrence of fragmented and fused predictions in variants/lineages with complex reassortment histories [28]. Fragmented predictions were generally observed in reassortant variants that derived their segments from three or more different parental viruses or in variants from second- and third-generation reassortment. In these cases, a reassortant found by GiRaF would display/be compatible with multiple different reassortment patterns at the same time. Fused predictions were mostly observed in second- and third-generation reassortment and in distinct reassortment events with very similar phylogenetic histories, resulting in false groupings of separate reassortment events. Thus, the exact patterns of segment exchange could not be resolved with certainty for some genomes and lineages based on GiRaF and ML analyses. Of the 8 reassortant lineages/clades where the reassortment patterns could be determined, 6 had their HA segments derived from one lineage and their NA segments from another, displaying a high level of HA-NA separation during reassortment (Fig. 3). None of the reassortant lineages were found to have arisen through reassortment of viruses originating from differing vaccine- and vaccine candidate-associated clades. On the other hand, four of the single reassortant genomes did contain segments derived from different vaccine and vaccine candidate clades (note arrows connecting reassortants to labeled vaccine clades in Fig. 4). Reassortment of segments from different vaccine-associated clades, especially when separation of HA and NA is observed, indicates reassortment between viruses with likely distinct antigenic properties.

Thus, shortly following their formation, several variants of H3N2 intra-subtype reassortants were able to establish a successful spread in the human population at the same time. Although the length of reassortant spread was temporally limited, the spatial reassortment patterns involved variants with history of previous reassortment and potentially differing antigenic properties.

### Positions with specific amino acid patterns and entropy define globally spread lineages

**Fig. 5:**
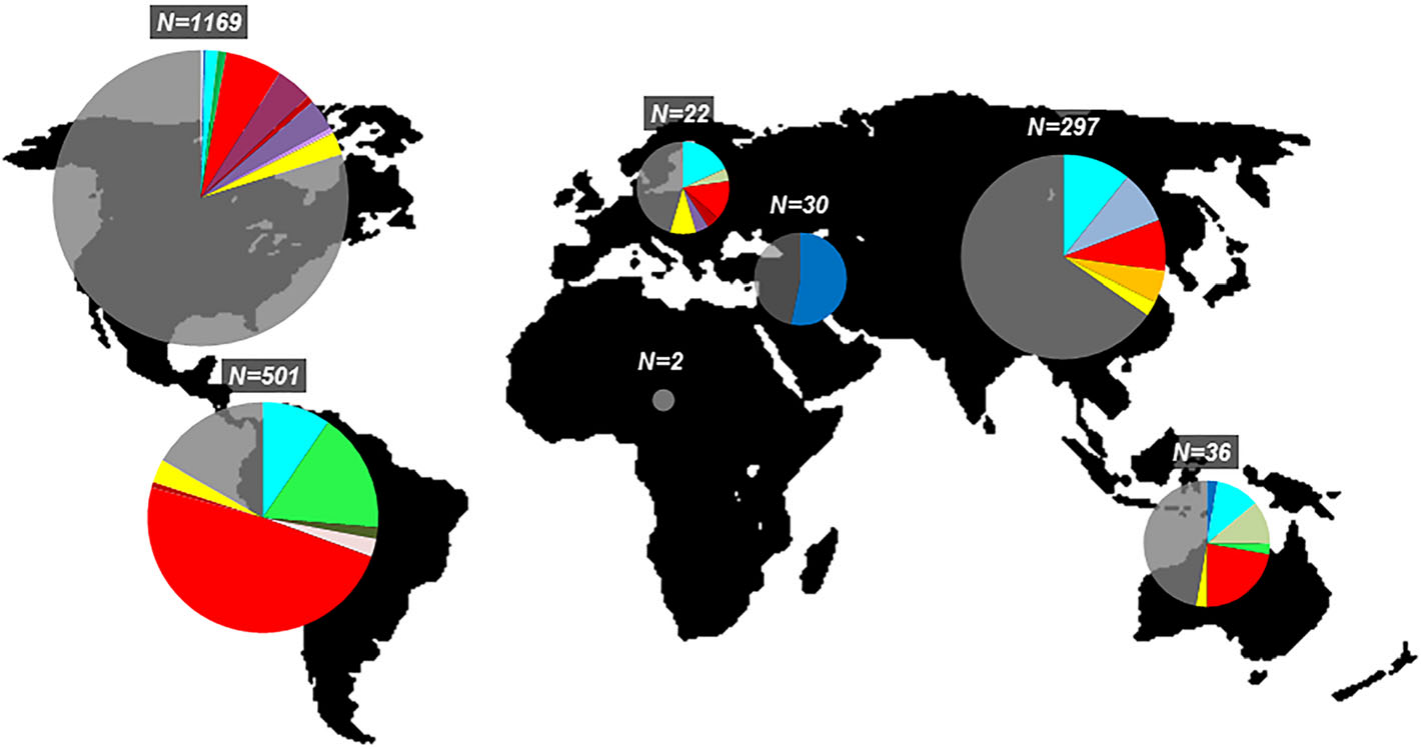
Global distribution of the H3N2 intra-subtype reassortants. Colors correspond to the reassortant lineage/clade colors in the maximum likelihood tree (see Fig. 1). Unique reassortants are represented by *yellow*. Non-reassortant viruses are *transparent gray. N* denotes the total number of genomes sampled from a region. Circle size is arbitrary for clarification purposes and is not proportional to *N*

The 16 reassortant variants that established successful spread in the human population between 2008 and 2014 also displayed differences in their geographical distributions. Some reassortant lineages spread globally, while others were mainly found transmitting in specific regions of the world. In addition, certain regions of the world displayed a higher presence of influenza reassortant genomes than others, with the most notable differences between North America and South-Central America, with 20.2% and 83.2% of their genomes being reassortants, respectively (Fig. 5).

To investigate properties of the different reassortant clades/lineages, we divided them into groups, those spreading globally (Global) or locally, and the locally spreading ones into high (High Local) or low (Low Local) (Table 1, for group definitions see Methods, subsection: Amino acid and entropy analyses in global and local lineages). Investigation of differences in amino acid Shannon entropy, i.e., the amount of amino acid variation in a certain position, revealed positions significantly different between the global and local lineages. These positions were located in only two of the segments, HA and NA. Combining the NA results from both types of Global versus High Local comparisons (pairwise and grouped, see Methods), only position 151 was found to significantly vary in all three Global reassortants but not in the High Local ones (Table 4). This position was also found to be under positive selection pressure in the Global reassortant lineages but not in the High Local (Table 5). In addition, the number of ambiguous nucleotide positions and the number of sequences containing them was significantly higher in the Global lineages (*p* < <0.01 and *p* < <0.01, *F* test). In HA, position 215 had significantly higher entropy in two of the three Global reassortants compared to the High Local ones (Table 4). HA position 215 was not found to be under positive selection in any of the reassortant lineages; however, the adjacent position 210 was positively selected in the Global lineages (Table 5). There were no positions that significantly differed in their entropy and that were specific for a certain geographical region in any of the segments.

**Table 4:** Amino acid position differences between Global and High Local reassortant lineages

		Global reassortant lineage			High Local reassortant lineage						
					N America		SC America	Asia		Australia	M East^a^
	Segment/Position	*X* (Red)	*XII* (Dark Red)	*III* (Light Blue)	*XI* (Maroon)	*XIV* (Purple)	*VII* (Green)	*IV* (BlueD)	*XIII* (Orange)	*VI* (GreenL)	*II* (Dark Blue)^a^
Shannon entropy differences	NA/151	D/N/G	D/N/G	D/N	D	*D/*_*G*_	D	*D*	*D*	D	*D/N*
	HA/215	A/S	A/S	S	S	S	S	S	S	S	S
Amino acid differences Global vs. Local	PB2/81	I	I	I	M	M	M	M	M	M	M
	PB2/255	I	I	I	V	V	V	V	V	V	V
	PB2/392	D	D	D	E	E	E	E	E	E	E
Amino acid differences Local	PB2/221	S	S	A	S	S	A	A	A	A	A
	PB2/293	K	K	R	K	K	R	R	R	R	R
	PB2/353	K	K	R	K	K	R	R	R	R	R
	PB2/560	I/V	I/V	I/V	I	I	V	V	V	V	V

^a^Although assigned High Local by the criteria, it cannot with certainty be regarded as such due to low regional and temporal sampling. It was included for exploratory purposes only

*D/N* Position was not conserved in this reassortant lineage, and the Shannon entropy of the Global reassortants was not significantly higher in any of the pairwise comparisons

*D/*_*G*_ Only one of the reassortant genomes had a differing amino acid in this position. Shannon entropy of the Global reassortants was not significantly higher in any of the pairwise comparisons

*D* Position was conserved in these reassortant lineages, but the Shannon entropy of the Global reassortants was not significantly higher in any of the pairwise comparisons

**Table 5:** Amino acid positions under positive selection pressure in Global and High Local reassortant lineages

	Global		High Local		*II* ^a^	
Method	NA	HA	NA	HA	NA	HA
SLAC	151	210	–	–	–	–
FEL	151, 331	210, 505	–	277	–	–
FUBAR	151, 331	210, 155, 224, 296, 505	–	277	151	–

^a^Although assigned High Local by the criteria, it cannot with certainty be regarded as such due to low regional and temporal sampling. The lineage was thus analyzed separately

Examination of raw amino acids between the reassortant variants revealed consistent differences in only one of the segments: PB2. Here, positions were found where globally spreading reassortants shared amino acids that were not found in any of the locally observed variants (Table 4). PB2 was also the only segment in which positions were found with region-specific amino acids (Table 4). None of the positions in PB2 were under positive selection pressure. In addition, we did not observe any amino acids that differentiated high and low occurring reassortant variants from the same region.

Thus, the intra-subtype H3N2 reassortants that establish an effective spread within the human population may spread globally or may be confined to certain geographical regions. Our results indicate that variants with successful global spread share some common properties in amino acid positions located within the HA, NA, and PB2 segments.

## Discussion

Influenza virus intra-subtype reassortment is a common process shown to play an important role in the evolution and adaptability of this virus. Intra-subtype reassortment is capable of producing epidemiologically relevant variants with impact on virus spread, disease severity, antiviral resistance, and vaccine efficacy. Detailed analyses of 2091 H3N2 full genomes sampled from different regions of the world between 2008 and 2014 revealed an extensive presence of intra-subtype reassortment variants, comprising 39% of all the H3N2 genomes studied. The frequency of reassortment events during this period of time was estimated at 3.35%, meaning the majority of reassortant genomes circulating in 2008–2014 were derived from a few reassortment events that were followed by establishment and successful transmission of the reassorted viruses within the human population. Furthermore, the spreading reassortants were found to continue reassorting, giving rise to second- and even third-generation reassortant viruses. The frequency of this continued reassortment did not differ significantly from the baseline frequency of 3.35%, nor did it differ significantly between the reassortant lineages or lineages with no observed history of reassortment. The notion of a stable frequency of H3N2 intra-subtype reassortment, not affected by the geographic and evolutionary origins of the viruses or by the presence of previous reassortment, would imply that this process occurs steadily and continuously for at least this subtype. The correlation between the number of reassortment events and the total number of genomes within a lineage might also suggest that the number of reassortment events in an influenza season will depend on the number of infected individuals during the epidemic; i.e., the bigger the epidemic, and the more viruses that circulate in the human population, the more reassortment events may occur. However, this possibility needs to be investigated further with more detailed and focused studies, as the number of sampled genomes in this study might not accurately reflect the number of infected individuals in an epidemic.

Sampling bias of influenza genomes is a known problem that may skew results if left unaccounted for. Indeed, the dataset used in this study suffered from the same issue, with few genomes sampled from Africa and many from North America. Temporal sampling differences may also exist, and the persistence of different lineages through several influenza seasons [18, 19] may induce another potential bias to time-based analyses. Because of this, region- or season-specific reassortment frequency was not estimated. Instead, lineage-specific and a total frequency of reassortment events were calculated and showed no significant differences, thus indicating that these estimates were not affected by uneven sampling. This was further confirmed by estimating the frequency of reassortment events in datasets that were adjusted for sampling bias towards North America. On the other hand, estimating the overall fraction of reassortant genomes may depend on geographical sampling. The size of this fraction in an epidemic will depend on the success of reassortant variant transmission following its creation, a transmission that may be affected by vaccine efficacy, host genetics and natural immunity, geography, or intrinsic properties and fitness of the reassortant variant itself [52].

Indeed, our results show that reassortant lineages, and thus the number of circulating reassortant genomes, may be unevenly distributed throughout the world. Therefore, our overall average estimate of the fraction of reassortant genomes in the dataset as a whole may change, as more genomes become available from underrepresented geographical regions. Confirming this notion, the fraction of reassortants in the datasets adjusted for sampling bias towards North America was significantly different from the fraction estimated in the full dataset and MrBayes subset analyses. Thus, making a clear distinction between the reassortment event frequency and the fraction of reassortant genomes becomes very important, as these two measures represent different properties of intra-subtype reassortment, and as they are affected very differently by sampling, geography, and time. Variations in the reassortment estimates have been observed when viewed from seasonal and regional perspectives, with sometimes unexpectedly high differences [17, 26]. These variations may be true reflections of temporal changes within smaller geographical regions, or they may be skewed if they fail to take into account the existence of lineages through several influenza seasons. Whatever the case, these limitations result in lack of power to reveal the general characteristics of H3N2 intra-subtype reassortment. Our study of full genomes 2008–2014 collected from most regions of the world revealed a stable reassortment frequency of 3.35%, where 23% of reassortment events resulted in creation of variants capable of establishing successful spread in the human population.

This study used a combination of MCMC and ML segment and full genome analyses for detection of reassortment. Generally, constructing full genome influenza H3N2 phylogenies may be misleading, as concatenation of reassorted segments results in creation of full genome recombinant sequences, which may impact the inferred tree topology and branch lengths. On the other hand, genomes that share the same reassortment/recombination patterns will cluster together in a full genome tree with higher confidence, drawn together by the strong signal of their common reassortment/recombination ancestry. Furthermore, full genome trees will display better node support due to additional genetic information gained through segment concatenation [53–55]. These features will allow for a more confident reassortant lineage definition, which is especially difficult on some H3N2 segment trees due to their low genetic diversity.

However, it is important to remember that the full genome trees cannot be used for analyses of lineage spatial and temporal relationships, only for refinement of reassortant lineage identification, as utilized in this study. The combined MCMC and ML approach thus allowed us to more confidently estimate H3N2 reassortment events. However, some events might still have been missed due to the difficulty of reassortment identification between strains of very similar sequences. In addition, incongruence can also be caused due to incomplete segment lineage sorting, resulting in an overestimation of reassortment. Using all 8 segments in the GiRaF analyses aided in the sensitivity of the reassortment detection, but the above caveats still highlight the need for development of better methodologies in this area of research. In our analyses these limitations were especially evident in determination of the exact patterns of segment exchange in the already detected reassortants. In fact, none of the approaches, including ML, RDP, GiRaF, BEAST-TMRCA, or BEAST-reassortment traits [10, 28, 30, 48], were specific, sensitive, or powerful enough to reveal all the specific segment exchange patterns. The ability to track reassortment patterns of the HA and NA segments is of particular importance, as this can result in rapid antigenic changes and affect vaccine efficacy.

Among the lineages in which we were able to determine patterns of segment reassortment, we did observe frequent exchange involving the HA and NA segments and, more importantly, segment exchange between viruses originating from different vaccine clades. Although genetic clustering does not always resolve viral antigenic differences (viruses within the same clade can have different antigenic properties), increasing genetic distance does result in accumulation of antigenic distances, and viruses clustering around different vaccine genomes in a phylogenetic tree will most likely display differing antigenic properties [56–58]. Intra-subtype reassortment of H3N2 variants with different antigenic profiles has been observed before and resulted in a major H3N2 intra-subtype antigenic shift and vaccine failure [23]. In our data, we found four reassortment variants with segment exchanges involving four major vaccine and vaccine/candidate clades observed between 2008 and 2014. Three of those variants gained their HA and/or NA segments from viruses belonging to the previous vaccine clades. If the number of infections or vaccine efficacy from these previous seasons was high, the general population would already be protected from these variants, and their transmission within the human population would be limited. Indeed, all four variants represented single reassortment events without further spread in the population. This concurs with vaccine efficacy results for the period between 2008 and 2014, which show that the vaccines for these seasons were close to their expected efficacy of 50–60% [59]. On the other hand, none of the spreading reassortant lineages from our study seemed to have originated by segment exchange between different vaccine clades, and the lack of pre-existing immunity against these variants may have helped facilitate their spread.

Our results illustrate the impact vaccines may have on the evolution, diversity, and transmission of the influenza virus. In addition, the occurrence of reassortment between different vaccine clades involving segments of antigenic importance demonstrates the underlying potential of influenza intra-subtype reassortment to induce swift antigenic change and highlights the significance of an effective influenza vaccine. It is difficult to speculate on what the low efficacy (19%) of the 2014/2015-season Texas/50/2012 vaccine [59] may lead to, but it is interesting to note that our 2014 sequences did make up a reassortant lineage of their own. Whether the vaccine efficacy was affected by the appearance of this reassortant lineage, or the spread of the reassortant was enabled by the composition of the vaccine itself, remains to be uncovered. Future analyses of vaccination rates versus timing of the reassortant spread could help shed light on this question, but it is also possible that no relation between this reassortant lineage spread and vaccine or its efficacy exists. Our results highlight, nevertheless, the importance of constant surveillance and tracking of reassortment variants, as their frequent formation and spread presents a continuous threat.

The spreading reassortant variants found in our study formed lineages whose existence overlapped through time and that were able to coexist both in single and multiple influenza seasons. Measuring the time between a reassortant lineage’s TMRCA and the year of its sampling allowed for a rough estimate of the lag time between reassortment and its detection on the population level. Some reassortants were identified very shortly after their formation, highlighting their rapid transmission potential but also demonstrating that their early detection is possible. Other reassortants showed considerable lag, up to 1.7 years, between their formation and detection at the population level. This indicates that reassortant variants may not initially be capable of widespread transmission but may exist at a very low level in the human population. Through genetic drift, and aided by the increased rate of adaptive amino acid replacements, these can eventually attain the capability of rapid and extensive spread [19, 25].

The characteristics of reassortant lineage spread varied greatly. Some variants were able to spread globally while others stayed mainly local, and some variants were able to spread more successfully in a population than others. These differences presented an opportunity to investigate the possible existence of specific features that would make a virus more fit to spread worldwide and to a high level. Significantly higher Shannon entropy was found in viruses from global reassortant lineages compared to those from local lineages, in both HA and NA. Higher variation would be expected in lineages that persist longer, as the global lineages in this study did on average. If random, this variation would not be expected to occur in the same amino acid positions across the globally spread lineages. This was, however, observed for two positions of the HA and NA segments. Position 215 of HA is a previously established antibody binding site within the antigenic epitope D of influenza virus and has also been described as a part of a human T cell epitope [60, 61]. In NA, position 151 has been shown to play a role in catalytic activities and receptor binding properties of the virus, as well as drug susceptibility to zanamivir [62, 63]. Higher entropy in certain key amino acid positions of globally transmitted reassortant lineages might be an inherent property of these viruses, resulting in a greater plasticity and thus a greater ability to adapt and overcome host and environmental global differences. Our results showed that NA position 151 was under strong positive selection in the globally spread lineages, while no such pressure existed in the local lineages. This may indicate greater adaptation pressure on this position that results in rapid fixation during local spread. Another potential source of the positive selection in this position could be extensive Madin-Darby canine kidney (MDCK) epithelial cells passaging, which has been suggested to increase variability of pH1N1 and possibly H3N2 NA 151 [64, 65]. Although this possibility would need to be investigated further, we found no significant difference between the number of passaged viruses in local and one proposed global lineage in our data (*t* test, *p* > 0.05), suggesting that MDCK passaging was probably not responsible for the observed difference in variability between these lineages in this specific position. However, we cannot exclude the possibility that the passaging history of the other three Global lineages differed from the one examined here. Further suggestive of the notion of greater adaptability was the significantly higher frequency of ambiguous nucleotide positions and sequences with ambiguous nucleotide positions throughout the HA and NA segments of globally spreading reassortants. This finding, which would need to be confirmed in a controlled sequencing setting, would indicate that these viruses from the start had more existing evolutionary paths available for adaptation due to their higher intra-population diversity. No positive selection in the HA position 215 indicated that it might not play as great a role in this process as the position 151 in NA.

Interestingly, looking at the High Local reassortant lineage *II,* we notice somewhat higher variability of the NA position 151 compared to the other local reassortants, as well as positive selection pressure and a higher proportion of viruses with ambiguous nucleotide sites. Further analyses including more genomes from 2014 and 2015 will reveal whether this reassortant lineage, found mainly in our samples from Kuwait, indeed is local or if it is spreading globally. The observed higher entropy, selection, and presence of ambiguous nucleotides indicate, however, that this reassortant lineage might, at this point, be found throughout the world. Our results also indicated a possible role of certain PB2 amino acids in the regional and worldwide spread of the reassortant variants. Although we still lack information on the exact role these amino acids play for the influenza virus in general, other variations within PB2 have been shown to play an important role in replication effectiveness, virulence, and fitness of influenza viruses [66, 67]. The specific amino acid position properties of the globally spreading reassortant variants could thus be used for tracking and, more importantly, prediction of future influenza spread, aiding in predictions of vaccine efficacy and providing information crucial for construction of future influenza vaccines.

## Conclusions

In summary, we show that the intra-subtype reassortment of influenza H3N2 is a frequent and dynamic process, displaying layers of properties and events that can occur and exist at the same time. The non-discriminatory manner of influenza reassortment is reflected by its occurrence within and between the lineages/clades of H3N2, regardless of the viruses’ geographical or evolutionary origins or their previous history of reassortment. Unlike the occurrence of reassortment events, the establishment and the spread of a reassortant variant may not be an entirely random process. Many factors can come to play a role in this, such as viral, host, and environmental elements. These may not be mutually exclusive but may have different degrees of effect in different scenarios. Additionally, given the high frequency of H3N2 intra-subtype reassortment, the appearance of inter-vaccine clade variants comes as no surprise. The possible rapid change of the antigenic properties of these reassortants may affect both disease severity and vaccine efficiency, and it highlights the importance of detailed surveillance as well as a further understanding of the process of intra-subtype influenza reassortment.

## Additional files

Additional file 1: a) Nested PCR primers and b) universal and segment-specific primers. (XLSX 16 kb)
Additional file 2: Reference sequence accession numbers: a) IVR and IRD and b) GISAID. (XLSX 181 kb)
Additional file 3: Figures of separate segment ML phylogenetic trees. All genomes analyzed are included. The reassortant lineages are colored as in Fig. 1. The trees are expanded so that taxa names are visible. (PDF 795 kb)
Additional file 4: Separate segment and full genome ML phylogenetic trees in machine readable (Newick) formats (9 sheets). (XLSX 267 kb)
Additional file 5: BEAST XML files and a table of BEAST run seeds. (XLSX 407 kb)
Additional file 6: Reassortant lineage passaging information. (XLSX 14 kb)

## Abbreviations

AFRIMS: Armed Forces Research Institute of Medical Sciences
AIC: Akaike information criterion
aLRT: Approximate likelihood-ratio test
BEAST: Bayesian Evolutionary Analysis Sampling Trees
CI: 95% confidence interval
DoS: Department of State
FEL: Fixed Effects Likelihood
FUBAR: Fast Unconstrained Bayesian Approximation
GiRaF: Graph incompatibility-based Reassortment Finder
GISAID: Global Initiative on Sharing All Influenza Data
GMRF: Gaussian Markov random field
GTR + I + Γ: General time reversible with empirically estimated proportion of invariant sites and gamma distribution of among-site variation, 4 categories
HPD: Highest posterior density intervals
IRD: Influenza Research Database
IUPAC: International Union of Pure and Applied Chemistry
IVR: Influenza Virus Resource
M East: Middle East
MCMC: Markov chain Monte Carlo
MDCK: Madin-Darby canine kidney (epithelial cells)
ML: Maximum likelihood
N America: North America
NAMRU-6: Naval Medical Research Unit 6
NHRC: Naval Health Research Center
NNI: Nearest neighbor interchanges
RDP: Recombination Detection Program
SC America: South and Central America
SLAC: Single Likelihood Ancestor Counting
SPR: Subtree pruning and regrafting
TMRCA: Time of the most recent common ancestor
WRAIR: Walter Reed Army Institute of Research
