# Supplementary figures and images for: Frequency of influenza H3N2 intra-subtype reassortment: attributes and implications of reassortant spread
Source: BMC Biol. 2016 Dec 29;14:117. doi: 10.1186/s12915-016-0337-3 (PMC5200972; doi:10.1186/s12915-016-0337-3)

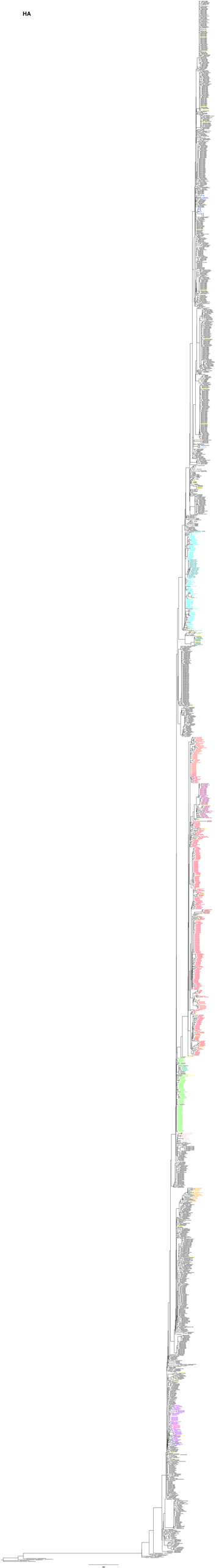

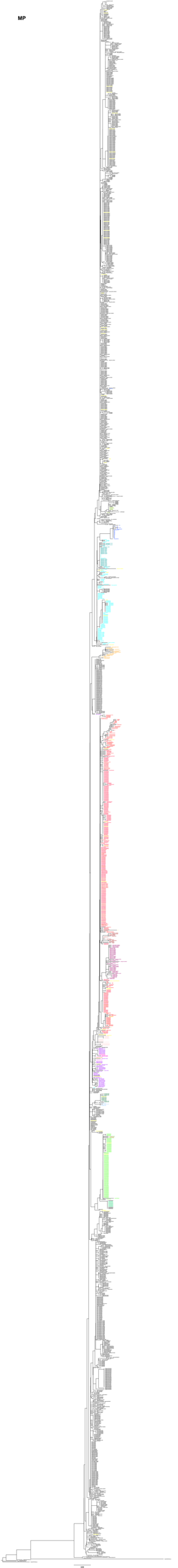

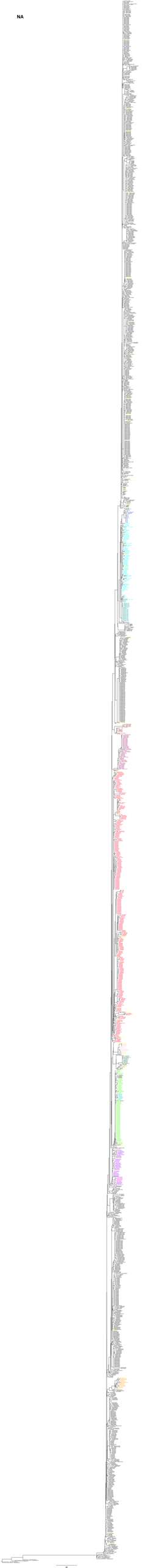

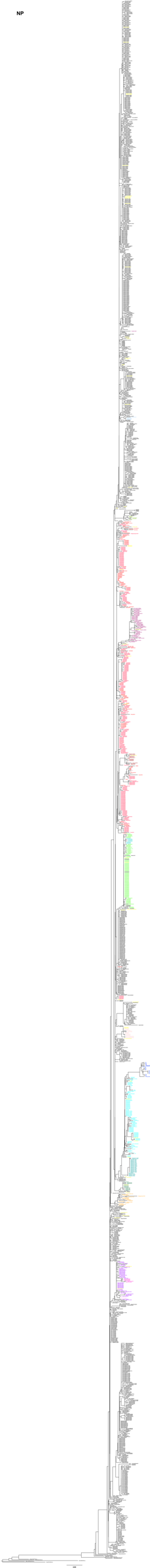

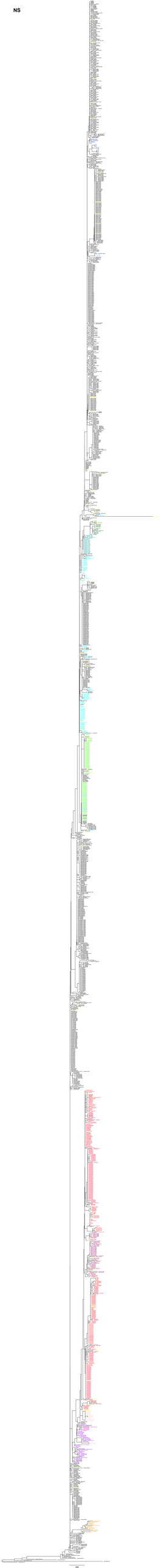

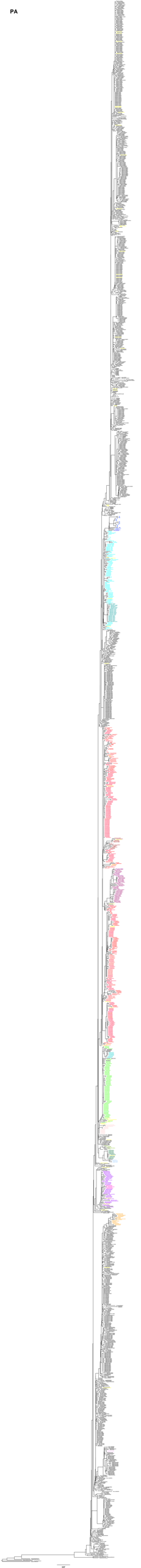

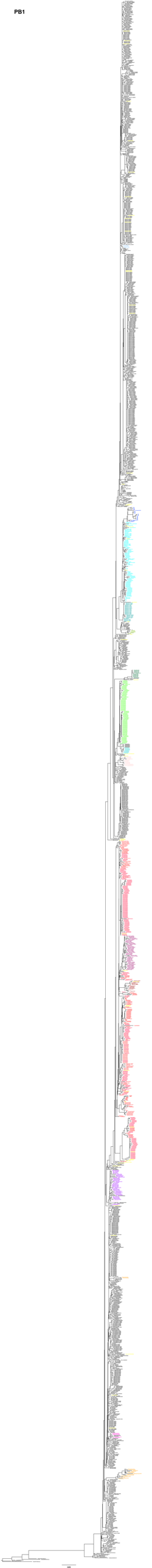

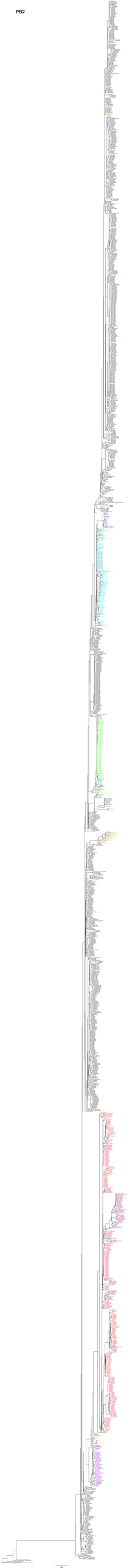

Supplement: Additional file 3: — Figures of separate segment ML phylogenetic trees. All genomes analyzed are included. The reassortant lineages are colored as in Fig. 1. The trees are expanded so that taxa names are visible. (PDF 795 kb) [file 12915_2016_337_MOESM3_ESM.pdf]
